# Functional Activation and Effective Connectivity Differences in Adolescent Marijuana Users Performing a Simulated Gambling Task

**DOI:** 10.1155/2015/783106

**Published:** 2015-01-26

**Authors:** Ashley Acheson, Kimberly L. Ray, Christina S. Hines, Karl Li, Michael A. Dawes, Charles W. Mathias, Donald M. Dougherty, Angela R. Laird

**Affiliations:** ^1^Department of Psychiatry, University of Texas Health Science Center at San Antonio, San Antonio, TX 78229, USA; ^2^Research Imaging Institute, University of Texas Health Science Center at San Antonio, San Antonio, TX 78229, USA; ^3^The Audie Murphy VA Medical Center, San Antonio, TX 78229, USA; ^4^Department of Physics, Florida International University, Miami, FL 33174, USA

## Abstract

*Background*. Adolescent marijuana use is associated with structural and functional differences in forebrain regions while performing memory and attention tasks. In the present study, we investigated neural processing in adolescent marijuana users experiencing rewards and losses. Fourteen adolescents with frequent marijuana use (>5 uses per week) and 14 nonuser controls performed a computer task where they were required to guess the outcome of a simulated coin flip while undergoing magnetic resonance imaging. *Results*. Across all participants, “Wins” and “Losses” were associated with activations including cingulate, middle frontal, superior frontal, and inferior frontal gyri and declive activations. Relative to controls, users had greater activity in the middle and inferior frontal gyri, caudate, and claustrum during “Wins” and greater activity in the anterior and posterior cingulate, middle frontal gyrus, insula, claustrum, and declive during “Losses.” Effective connectivity analyses revealed similar overall network interactions among these regions for users and controls during both “Wins” and “Losses.” However, users and controls had significantly different causal interactions for 10 out of 28 individual paths during the “Losses” condition. *Conclusions*. Collectively, these results indicate adolescent marijuana users have enhanced neural responses to simulated monetary rewards and losses and relatively subtle differences in effective connectivity.

## 1. Introduction

Marijuana use among adolescents is prevalent and increasing [1], and there is ample evidence that adolescents who use marijuana are at increased risk for developing psychiatric conditions including psychotic and affective disorders, as well as more severe substance use disorders [2–6]. Additionally, adult marijuana users who started in adolescence appear to show the most robust cognitive impairments [7, 8]. An emerging body of literature indicates that adolescent marijuana users have altered anatomy in prefrontal cortical and other forebrain regions [9–12] and corresponding functional differences while performing memory and attention tasks [13–18]. While it is unclear to what degree these outcomes are the result of marijuana use versus preexisting differences, there is evidence from animal models that adolescent exposure to tetrahydrocannabinol (THC), the primary active component of marijuana, can induce lasting structural brain changes accompanied by persistent cognitive and behavioral impairments [2, 19–21].

While reward processing has been studied rather extensively for many drug use disorders [22], to date relatively few studies have investigated neural substrates of reward processing in adolescent marijuana users. Adult marijuana users had increased striatal while anticipating rewards on the monetary incentive delay task, possibly indicating increased sensitivity to rewards [23]. Another study on adult marijuana users undergoing the monetary incentive delay task showed greater responses in reward circuitry in response to positive, but not negative, incentives, further suggesting that marijuana users may be more sensitive to rewards [24]. Adult marijuana users had decreased activity in anterior cingulate, medial frontal cortex, precuneus, superior parietal lobe, occipital lobe, and cerebellum following losses to negative feedback on the Iowa Gambling Task [25], also suggesting diminished response to negative incentives. However, behavioral data suggests that adolescent marijuana users are more sensitive to negative consequences, suggesting that this may be an opportune time to intervene behaviorally [26]. A better understanding of how reward cicutiry uitry functioning is altered in adolescent marijuana users may provide insight into mechanisms contributing to their use of marijuana or other substances.

In the present study, we sought to examine brain functioning in response to simulated monetary rewards and losses in adolescent marijuana users versus controls. Our methodology included examining alterations in neural circuitry using effective connectivity analyses. In contrast to more commonly used functional connectivity methods which simply investigate correlations between different brain regions, effective connectivity analyses offer the ability to investigate* causality* between regions, to probe subtle yet dynamic effects, such as differential patterns across clinical groups or developmental changes across the lifespan [27]. Given findings of marijuana users having altered activity in striatal, frontal cortical, and other regions during experiencing rewards and losses [23–25], marijuana users may be expected to show altered effective connectivity in these circuits as well. For instance, regions with increased activity in marijuana users may be less influenced by and/or have greater effects on other regions. Consequently, we expected connectivity in forebrain and other regions to be altered in adolescent marijuana users.

## 2. Methods

### 2.1. Participants

Fourteen marijuana using adolescents (11 males, 3 females) and 14 healthy control adolescents (11 males, 3 females) were recruited from the community through radio, newspaper, and television advertisements. Respondents to advertising completed an initial telephone interview to assess suitability for study participation, and potential participants were invited to the laboratory for a more comprehensive screening assessment of physical and psychiatric health, drug/alcohol use history, and intelligence. Psychiatric health was assessed using the Structured Clinical Interview for DSM-IV psychiatric disorders (SCID [28]) administered by trained research assistants and reviewed by a staff psychiatrist. Intelligence was assessed using the Wechsler Abbreviated Scale of Intelligence (WASI [29]). Family socioeconomic status was measured using the Four Factor Index of Socioeconomic Status (FFIS [30]). Self-reported impulsivity was assessed using the Barratt Impulsiveness Scale (BIS-11 [31]). Marijuana users were required to use marijuana 5 or more days per week.

Exclusionary criteria included physical or neurological conditions that would interfere with task performance, DSM-IV Axis I psychiatric disorder (other than cannabis use disorders for the user group including other substance use disorders), positive alcohol or drug screen (other than THC for the user group), or IQ < 80. All participants were between 15 and 19 years old. Written informed consent was obtained prior to study participation. The experimental protocol was approved by the Institutional Review Board of The University of Texas Health Science Center at San Antonio (UTHSCA).

### 2.2. Experimental Procedure

All participants completed two days of testing. Each day participants provided expired-air samples to screen for recent alcohol use (AlcoTest 7110 MKIII C, Draeger Safety Inc., Durango, CO) and urine samples to screen for recent drug use (THC metabolite, cocaine, benzodiazepines, opiates, and amphetamines; Panel/Dip Drugs of Abuse Testing Device, Redwood Biotech, Santa Rosa, CA). Participants in the user group were instructed not to smoke marijuana after midnight the night before testing sessions. On the first visit, participants completed questionnaires and interviews to verify study eligibility and obtain demographic and other pieces of information. On the second visit, participants underwent magnetic resonance imaging (MRI) on a research-dedicated Siemens 3 T MRI (Siemens, Munich, Germany) with a 12-channel head coil at the UTHSCA Research Imaging Institute.

### 2.3. Win/Loss Task

A block design task based on a similar paradigm used by Hariri and colleagues [32] was used to study neural activity associated with simulated monetary gains and losses. In this task, participants were presented with 6 blocks of 10 trials in which they were required to guess whether a simulated coin flip would be “heads” or “tails” interleaved; the coin flip blocks were alternated with 6 blocks of perceptual motor control trials. All trials were separated by a 0.2 s intertrial interval. On coin flip trials, participants were shown an image of coin head and tail for 1.9 s with the instruction “Please guess.” Participants were instructed that they would win 1 dollar for every correct guess and lose 1 dollar for every incorrect guess. However there was no actual coin flip occurring, and the task was programmed so that participants had an equal number of wins and losses regardless of what they guessed. Participants responded via button press on an MRI compatible response device and were then shown the predetermined outcome of either a win (the same coin face as guessed and the text “You Win $1”) or loss (opposite coin face as guessed and the text “You Lose $1”) for 0.9 s. On half the blocks of guessing trials, participants were given feedback that they won on 8 out of 10 trials (“Wins”). For the remaining blocks of guessing trials, participants were given feedback that they lost on 8 of 10 trials (“Losses”). During the perceptual motor control trials, participants were shown 2 blank coins with instructions to press either the left or the right coin. Participants were given feedback for correct or incorrect responses.

### 2.4. Imaging Acquisition

Functional imaging used a gradient-echo, echo-planar sequence, acquiring continuous 43 slices parallel to the anterior commissure-posterior commissure (AC-PC) plane (repetition time/echo time [TR/TE] = 3000/30 ms, 1.72 × 1.72 × 2.6 mm, and field of view [FOV] = 220 mm). For anatomical reference, a 3D high resolution T1-weighted series was acquired (TR/TE = 2000/2.83 ms, flip angle = 13°, 0.8 × 0.8 × 0.8 mm, and FOV = 256 mm).

### 2.5. Analysis of fMRI Data

Analysis of functional images was carried out using FSL (FMRIB's Software Library, http://fsl.fmrib.ox.ac.uk/fsl/fslwiki/ [33]). Prior to statistical modeling, data were motion corrected with MCFLIRT [34], nonbrain tissue was removed [35, 36] and spatially smoothed with a Gaussian kernel of FWHM 5 mm, and a high-pass temporal filtering was applied (Gaussian-weighted least-squares straight line fitting, with sigma = 50.0 s). Time-series statistical analysis was carried out using local autocorrelation correction [37, 38]. Registration to high resolution and/or standard images was carried out using FLIRT [39]. Higher-level analysis was carried out using FLAME (FMRIB's Local Analysis of Mixed Effects) with a mixed-effects model [37, 38] to generate *z* statistical images contrasting the “Win” versus control conditions and “Loss” versus control conditions. Contrasts for all subjects and for marijuana users versus controls were generated using conservative cluster thresholds (corrected *P* < 0.01, *z* ^3^ 2.3 [40]). The users versus controls contrasts were further thresholded with a postcontrast mask from each corresponding contrast with all subjects combined to examine group activation differences only in regions showing robust task effects across all subjects. Regions of interest (ROIs) for unified structural equation modeling were selected from user versus control contrasts (see Table 3). To identify regions with robust differences in group activity, cortical and subcortical activations with 15 or more voxels were selected, and a 5 mm diameter sphere was drawn centered on the peak *Z* stat coordinates.

### 2.6. Unified Structural Equation Modeling

The effective connectivity of activated regions during “Wins” and “Losses” was assessed using the unified structural equation modeling (SEM) approach [41, 42]. The distinction between traditional SEM and unified SEM is the inclusion of additional variables that improve the temporal representation of fMRI data via multivariate autoregressive modeling. Voxel-wise fMRI time series were extracted from each ROI and averaged for each subject's data set. The data from each ROI were normalized to a mean of zero and a variance of one. Due to the strong autocorrelations present in fMRI time-series data, each ROI was represented in the SEM by two variables based on a multivariate autoregressive lag 1 model: one of the time series extracted from the data set and the other a delayed version [41]. Thus, to assess the interactions between two brain regions A and B, four variables were created with three possible paths representing all possible effects of region A loading on B, including both* contemporaneous* effects and any* longitudinal* (delayed) effects. This general procedure for examining the relationships between two variables was then extended to simultaneously model all interactions for all variables.

### 2.7. Exploratory Model Generation

Rather than rely on* a priori* hypotheses for constructing paths in SEM, we applied an exploratory approach that was previously developed for identifying data-driven connectivity models [43], similar to what other investigators have proposed [42, 44]. Structural equation modeling was carried out in Amos 19.0 (SPSS, IMB, Inc.). Modeling began with the null model, which specifies that all measured variables are uncorrelated (with exception that the lag variable must be causally linked to its nonlag variable). The working model evolves by using modification indices to identify candidate connections, and the best candidates are tested to determine which paths (including contemporaneous and longitudinal effects) result in the largest reduction in the root mean square error of approximation (RMSEA). The RMSEA was selected to be the primary fit criterion because it is not as sensitive to the effects of sample size as other criteria [45]. The candidate connection yielding the greatest RMSEA improvement is selected, including both contemporaneous and longitudinal paths, and added to the overall model. This procedure is repeated to stepwise generate a best-fit model of the data. After a satisfactory level of model fit has been reached (an RMSEA of 0.05–0.08 [46]), the model is refined through the application of Occam's Razor: a specification search is conducted to remove loadings between variables that worsened or did not improve the key fit statistic of RMSEA in favor of parsimony.

Four models were independently generated using this technique; each model represents the best-fit model for each group and condition. Models were systematically compared using the procedure outlined in Figure 4. In addition, a “hybrid” model was created for each condition, comprising of all paths observed in either of the models* controls* or* users* to compare fit statistics across data sets for the* same model*. The fit statistics were computed for the hybrid model on data sets of each subject group independently. The hybrid model was then fit to the combination of both data sets, and the fit statistics were computed for this joint data set to assess controls and users simultaneously. To examine the influence of specific paths in the hybrid model across groups, we examined each path individually by constraining the path coefficients to be identical for the joint data and compute model fit statistics.

### 2.8. Assessing Goodness of Fit

Once a final model is identified, the goodness of fit is evaluated. Assessing model fit is not a simple process, and there exists no definitive way to determine how well a model represents an actual data matrix [47]. Moreover, there is no single statistical test that can be performed in order to identify that a given model is the correct model [48]. However, a large number of descriptive statistics have been developed to aid researchers in evaluating the adequacy or goodness of model relative to a given data (covariance) matrix. The procedure that we have developed is to evaluate final model fit using a set of multiple descriptive fit measures [43]. First, as stated above, we utilize the RMSEA as an index of model fit. Second, to examine overall model adequacy, the likelihood ratio chi-square statistic is used to test whether the discrepancy between the implied versus actual covariance matrices is statistically different [49]. Theoretical models that fit the given data perfectly have a chi-square value of zero. However, the chi-square statistic is sensitive to sample size, and while it can be an effective metric for effective connectivity models derived from positron emission tomography data, it is not as informative for fMRI models, given the increased temporal sampling and consistent degrees of freedom. Hence, we also consider both the Tucker-Lewis index (TLI) and the goodness-of-fit index (GFI), which range from values of 0 (no fit) to 1 (perfect fit), with values of 0.95 or greater indicating a good model [48]. Thus, for every final model, we compute fit statistics of RMSEA, chi-square, TLI, and GFI.

## 3. Results

### 3.1. Participants

The demographic characteristics, intelligence scores, self-reported levels of impulsivity, and current and lifetime recreational drug use histories reported by participants are summarized (Tables 1 and 2). Use of drugs other than marijuana in the user group was relatively limited; 10 out of 14 user subjects drank 1 or fewer alcoholic beverages per week and 9 out of 14 did not use any tobacco products. The groups did not differ in age, ethnicity, gender, intelligence (WASI), socioeconomic status (FFISS), or self-reported impulsivity (BIS-11 [31]).

### 3.2. Analysis of fMRI Data

**Figure 1:**
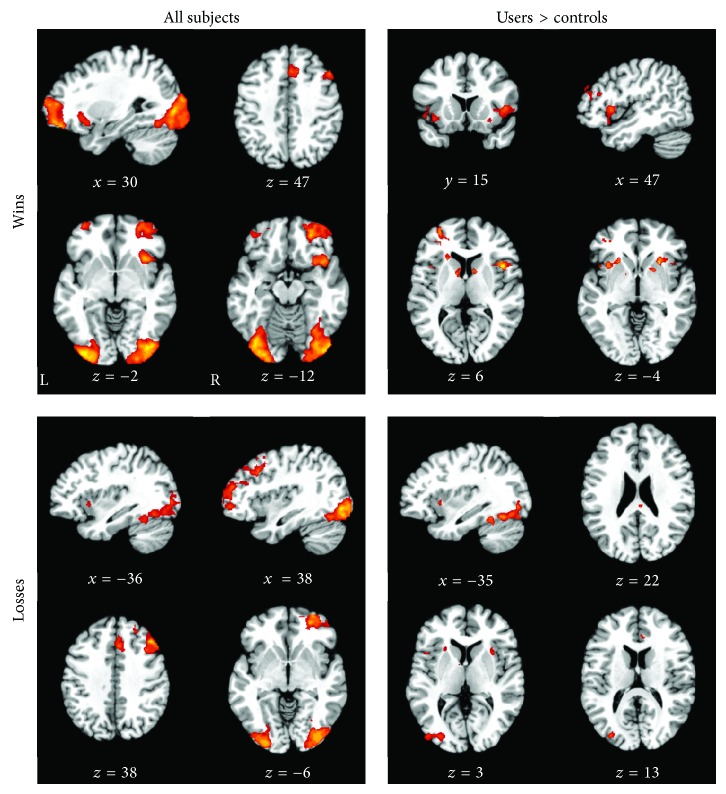


Across all participants, “Wins” were associated with large, widespread activations with peaks in the left cingulate, left superior frontal, right inferior frontal, and left middle frontal gyrus and bilateral declive (Table 3, Figure 1). “Losses” were similarly associated with large activations that peaked in the right middle frontal, cingulate, and middle occipital gyrus and left declive (Table 3, Figure 1). Within these regions, marijuana users had greater activity bilaterally in the middle frontal gyri, caudate, and claustrum during “Wins” and in the right middle frontal gyrus, right posterior and anterior cingulate, left insula, and bilateral claustrum and declive during “Losses” (Table 4, Figure 1). Controls did not show greater activity than users across either of the task conditions.

### 3.3. Effective Connectivity Analyses

**Figure 2:**
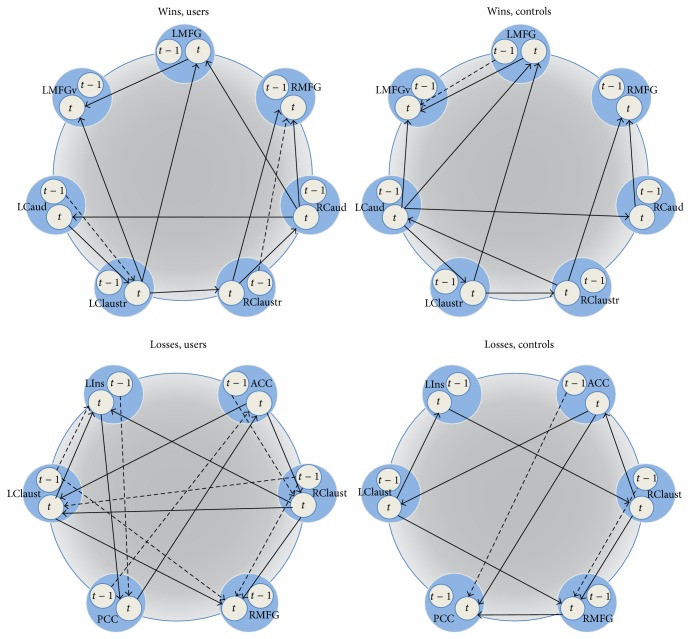


For “Wins,” network circuitry was evaluated across the left insula (LIns), anterior cingulate cortex (ACC), posterior cingulate cortex (PCC), bilateral claustrum (LClaust, RClaust), and left middle frontal gyrus (LMFG). For “Losses,” circuitry was examined in the bilateral and left ventral middle frontal gyrus (LMFG, RMFG, and LMFGv), bilateral caudate (LCaud, RCaud), and bilateral claustrum (LClaust, RClaust). Exploratory SEM identified best-fit models for “Wins” in controls (RMSEA = 0.063), “Wins” in users (RMSEA = 0.064), “Losses” in controls (RMSEA = 0.059), and “Losses” in users (RMSEA = 0.064) (Table 5, Figure 2). Reasonable model fits were obtained when controls and users data were tested on the reversed models, indicating that there were not any substantial network differences across groups (see S Table 1 in Supplementary Material available online at http://dx.doi.org/10.1155/2015/783106). However, we note that the controls fit the users data better than the users fit the controls data, which was likely due to the relatively parsimonious controls models.

**Figure 3:**
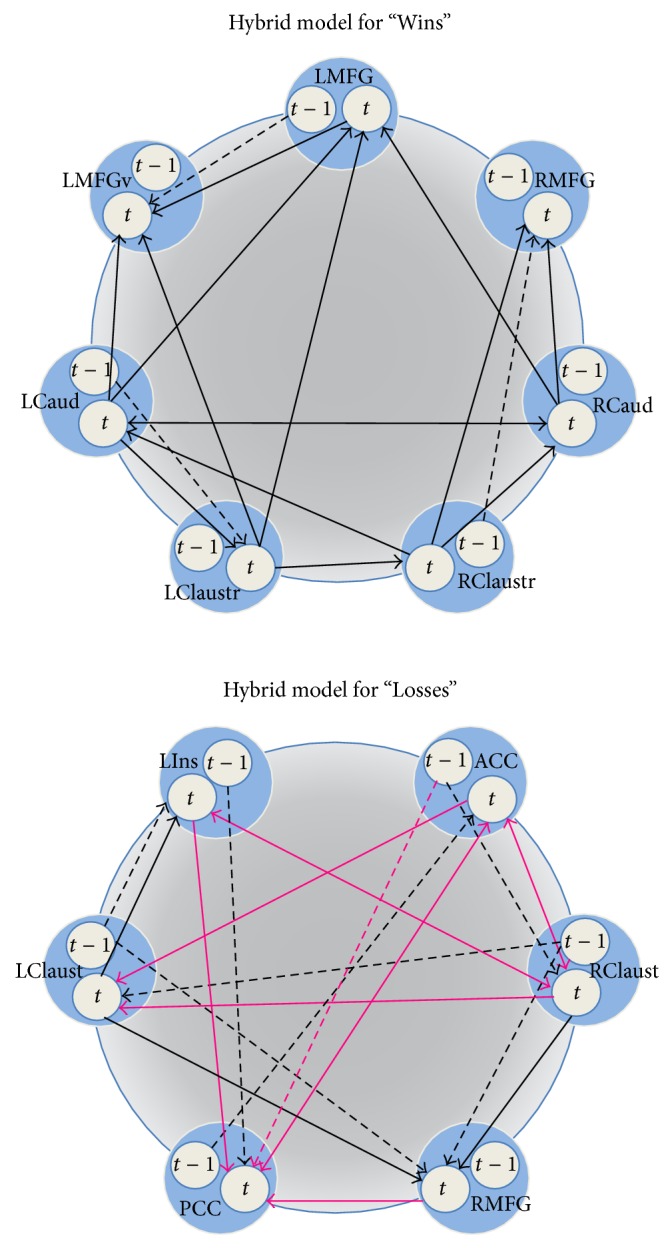


**Figure 4:**
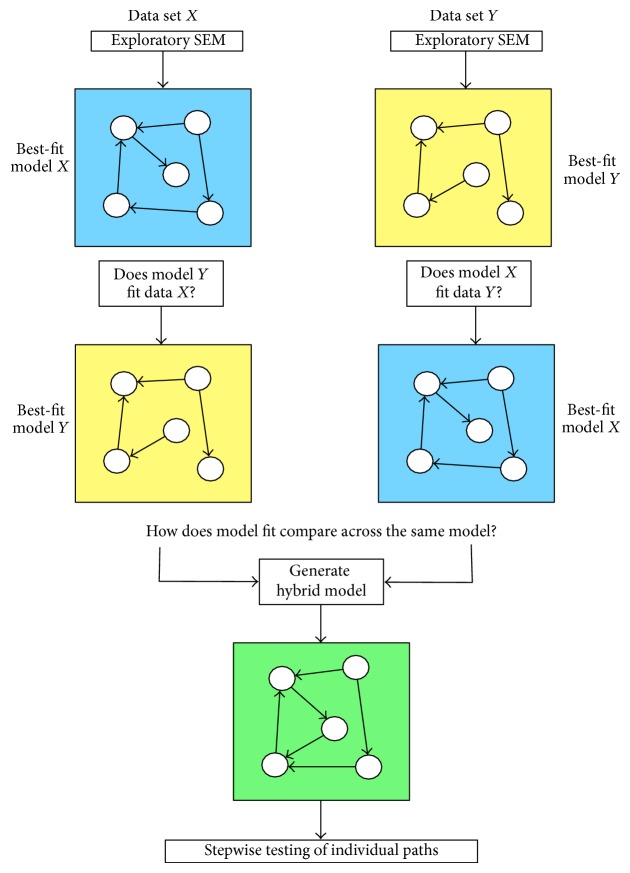


Additionally, both controls and users data were observed to fit the hybrid model well, including “Wins” in controls (RMSEA = 0.047), “Wins” in users (RMSEA = 0.052), “Losses” in controls (RMSEA = 0.050), and “Losses” in users (RMSEA = 0.056) (S Table  2). These results indicate that the overall networks could not be differentiated across subjects for both “Wins” and “Losses.” However, we next analyzed the individual paths in each hybrid model to assess more subtle differences across subject groups that did not affect the entire network. We found no differences across subject groups for “Wins” paths, regardless of which path was constrained. In contrast, we identified 10 paths of 28 total paths in the “Losses” hybrid model that significantly differed between users and controls; 9 of these were contemporaneous effects and one path (lag-ACC→PCC) was a longitudinal effect (S Table 3, Figure 3). Relative to controls, users showed weaker connectivity (1) between the anterior and posterior cingulate, (2) from the anterior cingulate to the right claustrum, (3) from the right medial frontal gyrus to the posterior cingulate, and (4) from the right claustrum to the left insula. Users also showed stronger connectivity from the right to left claustrum and altered connectivity between the left insula and posterior cingulate and anterior cingulate and left claustrum.

## 4. Discussion

As expected, this study confirms previous work showing that adolescent marijuana users have altered functioning in forebrain regions. Across all participants, “Wins” were associated with large activations that peaked in the cingulate, middle frontal, superior frontal, and inferior frontal gyri and declive and “Losses” were associated with similar large activations that peaked in the cingulate, middle frontal, and occipital gyri and declive. Within these regions, marijuana users demonstrated greater functional activation in the middle and inferior frontal gyri, caudate, and claustrum during “Wins” and greater activation in the anterior and posterior cingulate, middle frontal gyrus, insula, claustrum, and declive during “Losses.” These data indicate adolescent marijuana users show enhanced neural responses to simulated monetary rewards and losses. This may suggest that both positive and negative feedback may be helpful in behavioral modification of marijuana users.

Our finding of increased activity in the caudate during “Wins” suggests adolescent marijuana users may have increased responsiveness to rewards. However, our data failed to confirm prior work noting a decreased activity in cannabis using adults during negative feedback [24, 25]. In fact, we observed increased brain activity in marijuana users during “Losses” in structures associated with reward processing such as the insula, claustrum, and declive. Thus, our findings overall suggest that adolescent marijuana users may also exhibit enhanced sensitivity during negative feedback, consistent with findings from behavioral studies [24, 26]. Larger scale studies are needed to further elucidate the underlying reward mechanisms in adolescent marijuana users.

In contrast to our results using functional activation, we did not anticipate the results derived from our effective connectivity analyses, which showed little difference between overall network interactions for users and controls. These results indicate that, despite the activation differences, the overall functional circuitry in marijuana users is not substantially altered. In contrast, previous studies have found more profound connectivity differences among adult heavy users of marijuana as well as those dependent on cocaine, heroin, or methamphetamine [50–53]. For instance, adults with histories of more than 10 years of daily marijuana use show greater functional connectivity between the prefrontal and occipital parietal cortices while performing a cognitive interference task, possibly suggesting compensatory processes to overcome cannabis related cognitive impairments [50]. Additionally, cocaine dependent individuals had reduced resting state functional connectivity between the ventral tegmental area and striatum, amygdala and medial prefrontal cortex, and hippocampus and dorsal medial prefrontal cortex, and functional connectivity between the ventral tegmental area and striatum was negatively correlated with years of cocaine use [52]. Related, stimulant-dependent individuals had reduced resting state functional connectivity between the orbitofrontal cortex and dorsal medial premotor and cingulate cortices, similar to deficits observed in individuals with obsessive compulsive disorder [53]. Finally, heroin dependent individuals were observed to have reduced functional connectivity at rest between the right dorsolateral prefrontal cortex and left inferior parietal lobe which was also negatively correlated with years of use [51]. While overall the adolescent marijuana users in our study showed more modest connectivity differences, it is possible that individuals with longer total exposure to marijuana (i.e., heavier daily use or more chronic use) display more pronounced connectivity differences or that connectivity differences may be different whether subjects are at rest or while performing more cognitively challenging tasks.

In order to investigate more subtle connectivity differences, we further assessed the individual paths which revealed no differences between users and controls for the “Wins” condition; however, users and controls demonstrated significantly different causal interactions for 10 out of 28 total paths during the “Losses” condition. These data suggest that marijuana users may process negative feedback differently relative to controls, which if true may provide specific cognitive or behavioral interventions for use in therapy. Further research on the differences in causal interactions between adolescent marijuana users and controls is needed to clarify the connectivity differences.

Finally, the exploratory unified SEM approach offers an excellent opportunity for modeling effective connectivity in fMRI data. However, the results of this study may be limited in several ways. First, the sample size for this study was fairly modest. It is a possible that with additional subjects we would have had additional power to detect more substantive, network-wide connectivity differences. Related, our sample was mostly male and different activation and connectivity patterns could be present in female marijuana users. Additionally, while use of other drugs such as alcohol and tobacco was limited, we cannot rule out that other substances' use may have influenced our findings. In addition, there is a potential for the introduction of bias in our modeling due to the selection of ROIs based on the results of the activation analyses. This method for ROI selection may be considered as a form of “double-dipping.” Our rationale for this procedure was the lack of prior work in this domain, thereby decreasing our options for meta-analytically derived regions of interest. It is possible that with additional subjects our future work will include less-biased regional selection in a second data set [54, 55].

## 5. Conclusions

In summary, these results provide evidence that alterations in reward circuitry in this population cannot be determined as a network-wide phenomenon, which is not surprising given that subjects were neurologically and psychiatrically healthy aside from frequent marijuana use. However, by examining specific pathways during “Losses,” we identified that 1/3 of the total paths analyzed demonstrated differences between groups. These results may be related to the increased risk of future substance use disorders and other psychiatric conditions and may provide target neural connections to be monitored in these high-risk populations.

## Supplementary Material

The Supplementary Material provide additional information on the analytical steps involved with the unified structural equation modeling analysis. S Table 1 reports model fits when Controls and Users data were tested on the reversed models. S Table 2 reports Controls and Users data fits to the hybrid model. S Table 3 reports Controls and User data fits to individual paths in each hybrid model to assess more subtle differences across subject groups that did not affect the entire network.

## Figures and Tables

**Table 1:** Participant characteristics.

	Controls	Users
	*n* = 14	*n* = 14
Characteristics (mean ± SD)		
Age	17.3 ± 1.3	17.6 ± 1.0
WASI total IQ score	104.8 ± 9.4	99.6 ± 7.6
FFISS	37.8 ± 15.2	34.7 ± 17.2
BIS-11 attentional	13.9 ± 3.7	17.4 ± 4.2^*^
BIS-11 motor	20.1 ± 3.1	24.2 ± 4.0^*^
BIS-11 nonplanning	21.0 ± 3.4	23.8 ± 5.0
Gender (*n* (%))		
Male	11 (76%)	11 (76%)
Female	3 (24%)	3 (24%)
Ethnicity (*n* (%))		
African-American	2 (14%)	4 (29%)
Caucasian	5 (36%)	4 (29%)
Hispanic	7 (50%)	6 (42%)

^*^ *P* < 0.05.

**Table 2:** Marijuana and drug use summary.

	Controls	Users
Weekly drug use (mean ± SD)		
Marijuana (uses/week)	0	6.7 ± 1.5
Cigarettes (cigarettes/week)	0	5.4 ± 9.1
Alcohol (drinks/week)	0	3.7 ± 5.6
Lifetime drug use (# ever used)		
Marijuana (# ever used)	0	14
Stimulants	0	2
Opiates	0	3
Benzodiazepines	0	2
Hallucinogens	0	3

**Table 3:** Activations across all subjects.

Condition	Coordinates (mm)			Region	Brodmann area	Cluster size
	*X*	*Y*	*Z*			
Win	0	28	30	L cingulate gyrus	32	692
	−30	50	14	L superior frontal gyrus	10	566
	34	18	−4	R inferior frontal gyrus	47	537
	28	46	−10	R middle frontal gyrus	11	2256
	46	−80	−18	R declive	∗	3125
	−36	−78	−18	L declive	∗	2442

Loss	44	28	38	R middle frontal gyrus	8	2024
	2	28	30	R cingulate gyrus	32	533
	36	−82	−8	R middle occipital gyrus	18	2471
	−32	−84	−18	L declive	∗	1658

*P* < 0.01, *z* ≥ 2.3.

**Table 4:** Group activation differences.

Condition	Coordinates (mm)			Region	Brodmann area	Cluster size
	*X*	*Y*	*Z*			
Win	40	32	18	R middle frontal gyrus	46	212
	12	6	10	R caudate	∗	100
	−36	42	8	L middle frontal gyrus	10	226
	−10	4	6	L caudate	∗	56
	−24	24	0	L claustrum	∗	251
	26	16	−4	R claustrum	∗	390
	−32	42	−6	L middle frontal gyrus	11	68

Loss	48	6	36	R middle frontal gyrus	9	38
	4	−32	24	R posterior cingulate	23	23
	6	30	14	R anterior cingulate	24	17
	30	12	4	R claustrum	∗	121
	−44	12	2	L insula	13	32
	−24	18	−6	L claustrum	∗	43
	−40	−62	−18	L declive	∗	834
	32	−66	−18	R declive	∗	138

*P* < 0.01, *z* ≥ 2.3.

**Table 5:** Model fit statistics. Exploratory SEM was applied to identify best-fit models observed during “Wins” and “Losses” for marijuana users and healthy controls. Model fit was assessed using the root mean square error of approximation (RMSEA), chi-square statistic, degrees of freedom (dof), Tucker-Lewis index (TLI), and goodness-of-fit index (GFI).

	RMSEA	Chi-square	dof	TLI	GFI
Marijuana users					
“Wins” model	0.064	496.223	62	0.915	0.960
“Losses” model	0.064	285.827	36	0.898	0.974

Healthy controls					
“Wins” model	0.063	483.507	63	0.904	0.961
“Losses” model	0.059	285.764	42	0.908	0.972
