# Supplementary material for: Functional Activation and Effective Connectivity Differences in Adolescent Marijuana Users Performing a Simulated Gambling Task
Source: J Addict. 2015 Jan 26;2015:783106. doi: 10.1155/2015/783106 (PMC4321681; doi:10.1155/2015/783106)
Supplement: Supplementary file 1 — The Supplementary Material provide additional information on the analytical steps involved with the unified structural equation modeling analysis. S Table 1 reports model fits when Controls and Users data were tested on the reversed models. S Table 2 reports Controls and Users data fits to the hybrid model. S Table 3 reports Controls and User data fits to individual paths in each hybrid model to assess more subtle differences across subject groups that did not affect the entire network. [file 783106.f1.pdf]

S Table 1 (WAS6). Reversed Model Fit Statistics. “Wins” and “Losses” best-fit models generated using data corresponding one subject group was fit it to data corresponding to the other subject group to assess how similar the groups were in terms of network-wide effects. Model fit was assessed using the root mean square error of approximation (RMSEA), chi-square statistic, degrees of freedom (*dof*), Tucker-Lewis index (TLI), and Goodness-of-Fit index (GFI). Reasonable model fits were obtained, indicating that the overall networks *cannot* be differentiated across subjects for both “Wins” and “Losses”.

	**RMSEA**	**Chi-Square**	***dof***	**TLI**	**GFI**
**Marijuana Users**					
“Wins” Controls Model	0.083	805.311	63	0.856	0.937
“Losses” Controls Model	0.111	912.214	42	0.695	0.920
**Healthy Controls**					
“Wins” Users Model	0.071	596.317	62	0.876	0.952
“Losses” Users Model	0.089	515.824	36	0.790	0.954

S Table 2 (WAS 7). Hybrid Model Fit Statistics. Paths included in the best-fit models for Users and Controls were merged to create hybrid models for "Wins" and "Losses". Model fits were computed for each subject group to allow quantitative comparison of groups *on the same model*, including the root mean square error of approximation (RMSEA), chi-square statistic, degrees of freedom (*dof*), Tucker-Lewis index (TLI), and Goodness-of-Fit index (GFI). The data from both subject groups were observed to fit the hybrid models well, again indicating the overall networks *cannot* be differentiated across subjects for this task.

	**RMSEA**	**Chi-Square**	***dof***	**TLI**	**GFI**
**Marijuana Users**					
"Wins" Hybrid Model	0.052	311.433	55	0.943	0.975
"Losses" Hybrid Model	0.056	196.926	31	0.921	0.981
**Healthy Controls**					
"Wins" Hybrid Model	0.047	262.29	55	0.946	0.979
"Losses" Hybrid Model	0.050	160.17	31	0.934	0.983

S Table 3. (was 8) Comparison of Individual Paths Within Hybrid Models. To examine group differences for individual paths, each path coefficient was constrained to be identical for the joint data, and the resulting model fit statistic was computed. Decreases in RMSEA from unconstrained to constrained paths indicated that a particular path was differed between subject groups. This step-wise process was repeated for all possible paths in the hybrid models, yielding the RMSEA values listed above. While no differences were observed across subject groups for "Wins" paths, 10 paths in the "Losses" hybrid model were determined to significantly differ between Users and Controls, shaded and marked by an asterisk (*).

Hybrid "Wins" Paths	RMSEA	Hybrid "Losses" Paths	RMSEA
Unconstrained Paths	0.035	Unconstrained Paths	0.037
LCaud ⟶ RCaud	0.035	LClaust ⟶ LIns	0.037
lag-LCaud ⟶ lag-RCaud	0.035	lag-LClaust ⟶ lag-LIns	0.038
LCaud ⟶ LMFGv	0.035	lag-LClaust ⟶ LIns	0.043
lag-LCaud ⟶ lag-LMFGv	0.035	LClaust ⟶ RMFG	0.037
LCaud ⟶ LMFG	0.035	lag-LClaustr ⟶ lag-RMFG	0.039
lag-LCaud ⟶ lag-LMFG	0.035	lag-LClaust ⟶ RMFG	0.037
LCaud ⟶ LClaust	0.035	PCC ⟶ ACC	0.036*
lag-LCaud ⟶ lag-LClaust	0.035	lag-PCC ⟶ lag-ACC	0.037
lag-LCaud ⟶ LClaust	0.035	lag-PCC ⟶ ACC	0.037
LClaust ⟶ RClaust	0.035	lag-ACC ⟶ PCC	0.036*
lag-LClaust ⟶ lag-RClaust	0.035	RClaust ⟶ LClaust	0.036*
LClaust ⟶ LMFGv	0.035	lag-RClaust ⟶ lag-LClaust	0.037
lag-LClaust ⟶ lag-LMFGv	0.035	lag-RClaust ⟶ LClaust	0.038
LMFG ⟶ LClaust	0.035	RClaust ⟶ RMFG	0.037
lag-LMFG ⟶ lag-LClaust	0.035	lag-RClaust ⟶ lag-RMFG	0.038
LMFG ⟶ LMFGv	0.035	lag-RClaust ⟶ RMFG	0.048
lag-LMFG ⟶ lag-LMFGv	0.035	RClaust ⟶ LIns	0.037
lag-LMFG ⟶ LMFGv	0.035	lag-RClaust ⟶ lag-LIns	0.036*
RClaust ⟶ LCaud	0.035	LIns ⟶ PCC	0.036*
lag-RClaust ⟶ lag-LCaud	0.035	lag-LIns ⟶ lag-PCC	0.036*
RClaust ⟶ RCaud	0.035	lag-LIns ⟶ PCC	0.039
lag-RClaust ⟶ lag-RCaud	0.035	ACC ⟶ LClaust	0.036*
RClaust ⟶ RMFG	0.035	lag-ACC ⟶ lag-LClaust	0.036*
lag-RClaust ⟶ lag-RMFG	0.035	ACC ⟶ RClaust	0.037
lag-RClaust ⟶ RMFG	0.035	lag-ACC ⟶ lag-RClaust	0.036*
RCaud ⟶ RMFG	0.035	lag-ACC ⟶ RClaust	0.038
lag-RCaud ⟶ lag-RMFG	0.035	RMFG ⟶ PCC	0.036*
RCaud ⟶ LMFG	0.035	lag-RMFG ⟶ lag-PCC	0.040
lag-RCaud ⟶ lag-LMFG	0.035		
